# Supplementary figures and images for: EphrinB1 modulates glutamatergic inputs into POMC-expressing progenitors and controls glucose homeostasis
Source: PLoS Biol. 2020 Nov 30;18(11):e3000680. doi: 10.1371/journal.pbio.3000680 (PMC7728393; doi:10.1371/journal.pbio.3000680)

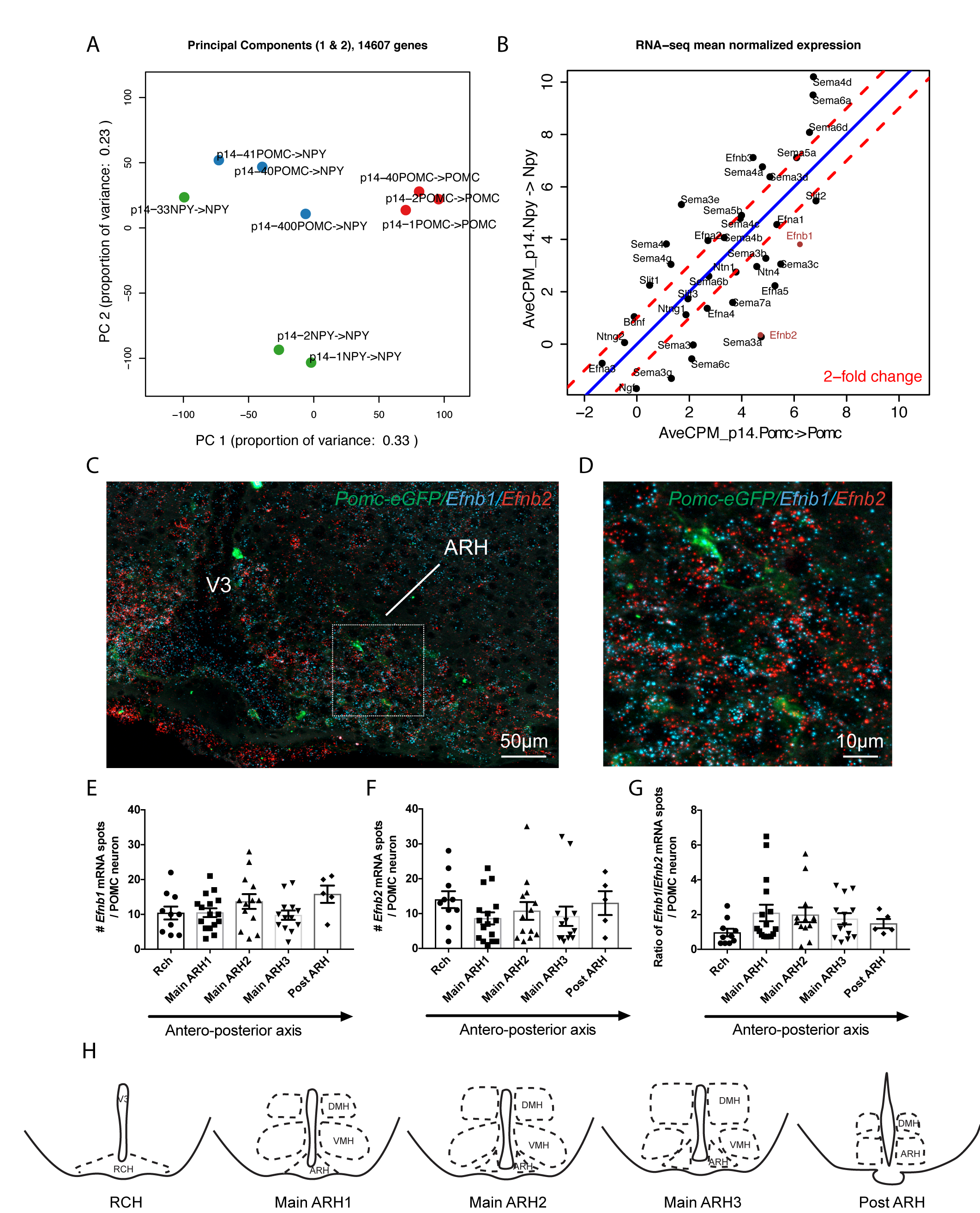

Supplement: S1 Fig — (A) PCA made on 14,607 genes. (B) Scatterplots comparing the expression of individual genes between Pomc->Pomc and Npy->Npy neuronal population at P14. (C) Microphotographs showing Efnb1 (blue) and Efnb2 (red) mRNA spots in POMC-GFP+ (green) neurons in the ARH of P14 male mice. (D) High magnification of the inset shown in (C). Quantification of the number of Efnb1 mRNA spots (E), Efnb2 mRNA spots (F) or Efnb1/Efnb2 mRNA spot ratio (G) in POMC-eGFP neurons in the entire thickness of the ARH of P14 male animals (n = 2 animals). (H) Schematic illustrating the subdivisions of the ARH used for the quantification in E, F, and G. Data are shown ± SEM. Statistical significance was determined using 1-way ANOVA (E–G). The underlying data are provided in S1 Data. ANOVA, analysis of variance; ARH, arcuate nucleus of the hypothalamus; DMH, dorsomedial nucleus of the hypothalamus; GFP, green fluorescent protein; NPY, neuropeptide Y; PCA, principal component analysis; POMC, proopiomelanocortin; RCH, retrochiasmatic area; SEM, standard error of the mean; VMH, ventromedial nucleus of the hypothalamus; V3, third ventricle. (TIF) [file pbio.3000680.s001.tif]

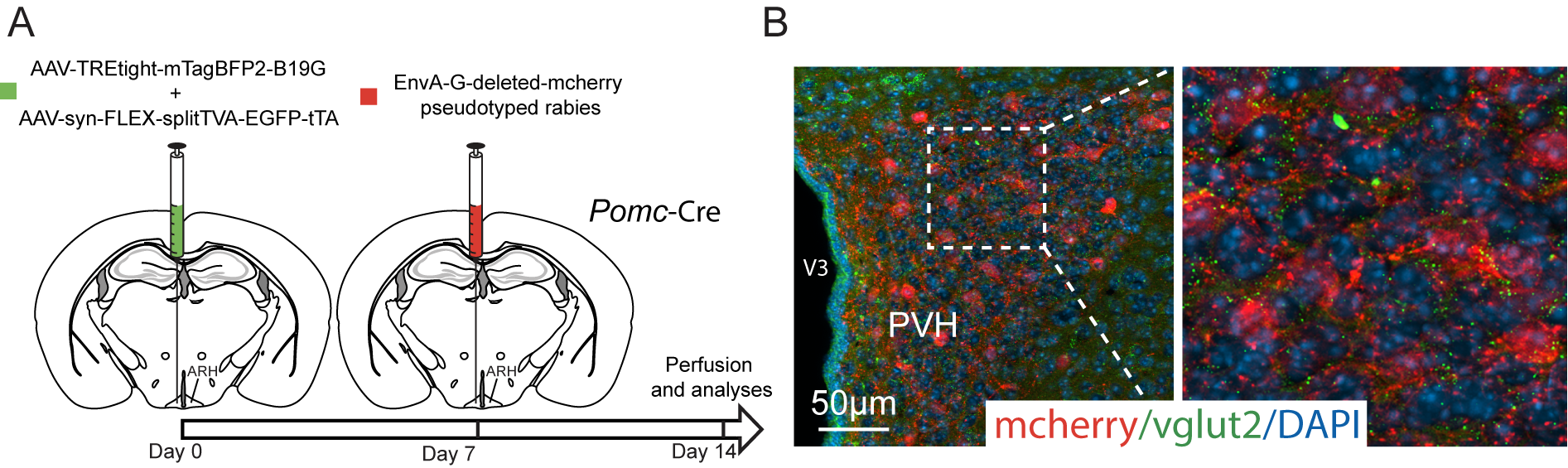

Supplement: S2 Fig — (A) Experimental approach. A mix of AAV-TREtight-mTagBFP2-B19G and AAV-syn-FLEX-splitTVA-EGFP-tTA was injected at day 0 in the ARH of 12-week-old Pomc-Cre male mice. Seven days later, mice received injection of EnvA-G-deleted-mcherry pseudotyped rabies. Animals were perfused 1 week later for further analyses. (B) Photomicrographs showing the co-localization of mcherry-positive cells (POMC inputs) with glutamatergic neurons of the PVH (vglut2 mRNA spots in green). DAPI counterstaining is shown in blue. ARH, arcuate nucleus of the hypothalamus; POMC, proopiomelanocortin; PVH, paraventricular nucleus of the hypothalamus; V3, third ventricle. (TIF) [file pbio.3000680.s002.tif]

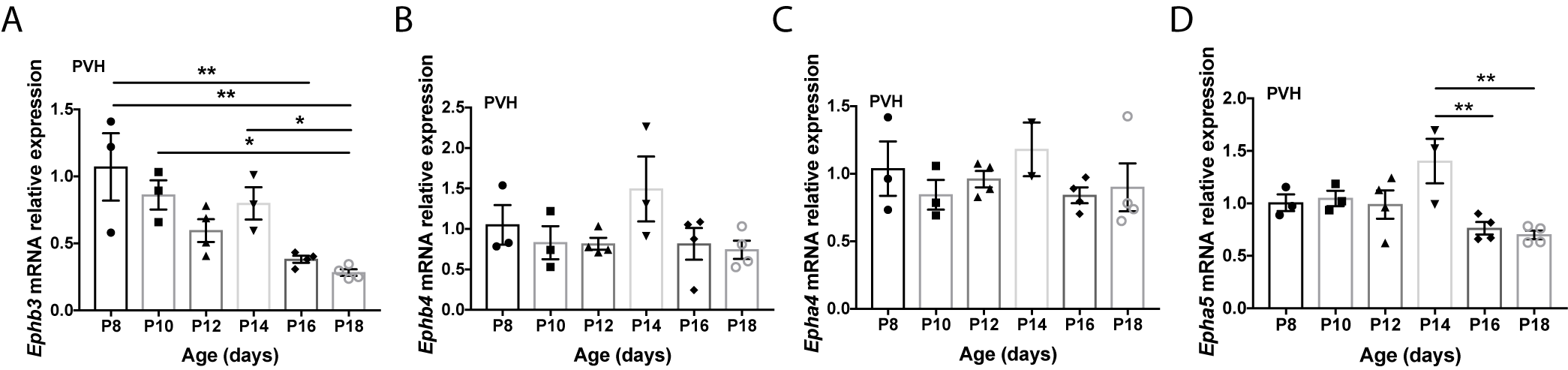

Supplement: S3 Fig — Quantification of Ephb3 (A), Ephb4 (B), Epha4 (C) and Epha5 (D) mRNA relative expression in PVH of P8, P10, P12, P14, P16 and P18 male pups (n = 2–4 pups/age). Data are shown ± SEM. Statistical significance was determined using 1-way ANOVA (A–D). *P ≤ 0.05 versus P12 (A), versus P14 (A), **P ≤ 0.01 versus P8 (A), versus P14 (D). The underlying data are provided in S1 Data. ANOVA, analysis of variance; PVH, paraventricular nucleus of the hypothalamus; SEM, standard error of the mean. (TIF) [file pbio.3000680.s003.tif]

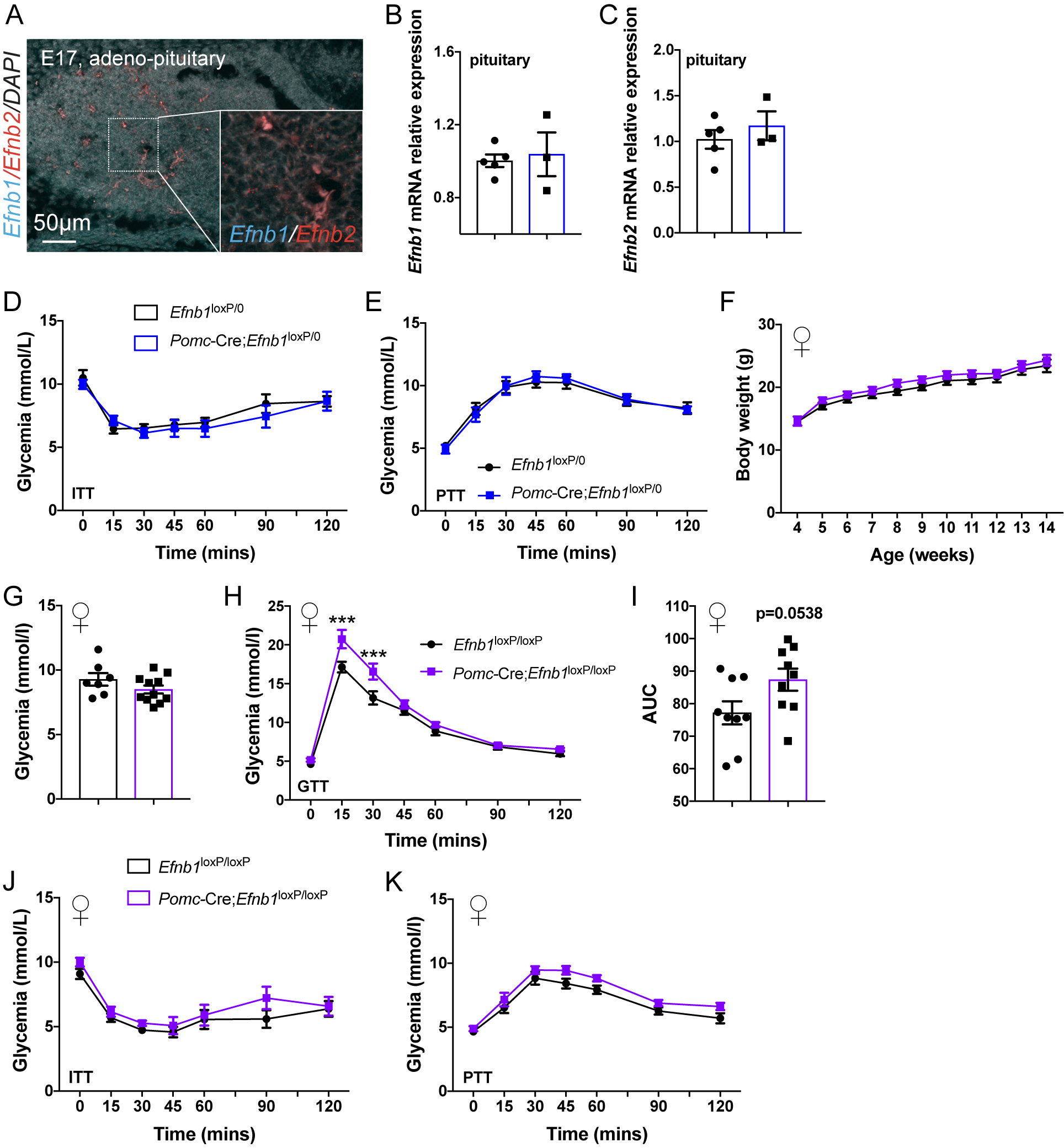

Supplement: S4 Fig — (A) Microscope image illustrating the expression of Efnb1 (blue) and Efnb2 (red) mRNA in the adeno-pituitary of E17 embryo. DAPI counterstaining is shown in white. (B) Efnb1 and Efnb2 mRNA relative expression in the pituitary of Efnb1loxP/0 and Pomc-Cre;Efnb1loxP/0 16-week-old male mice (n = 3–5/group). (D) Insulin tolerance test of 14-week-old male mice (n = 10–13/group). (E) Pyruvate tolerance test of 13-week-old male mice (n = 9–10/group). (F) Post-weaning growth curve of Efnb1loxP/loxP and Pomc-Cre;Efnb1loxP/loxP female mice (n = 8–11/group). (G) Basal glycemia of 8-week-old female mice (n = 7–11/group). (H) Glucose tolerance test of 8–9-week-old female mice (n = 9/group). (I) Area under the curve of GTT experiment. (J) Insulin tolerance test of 14-week-old female mice (n = 9/group). (K) Pyruvate tolerance test of 13-week-old female mice (n = 7–8/group). Data are shown ± SEM. Statistical significance was determined using 2-way ANOVA (D–F, H, J, K) and 2-tailed Student t test (B, C, G, I). ***P ≤ 0.001 versus Efnb1loxP/loxP (H). The underlying data are provided in S1 Data. ANOVA, analysis of variance; GTT, glucose tolerance test; ITT, insulin tolerance test; POMC, proopiomelanocortin; PTT, pyruvate tolerance test; SEM, standard error of the mean. (TIF) [file pbio.3000680.s004.tif]

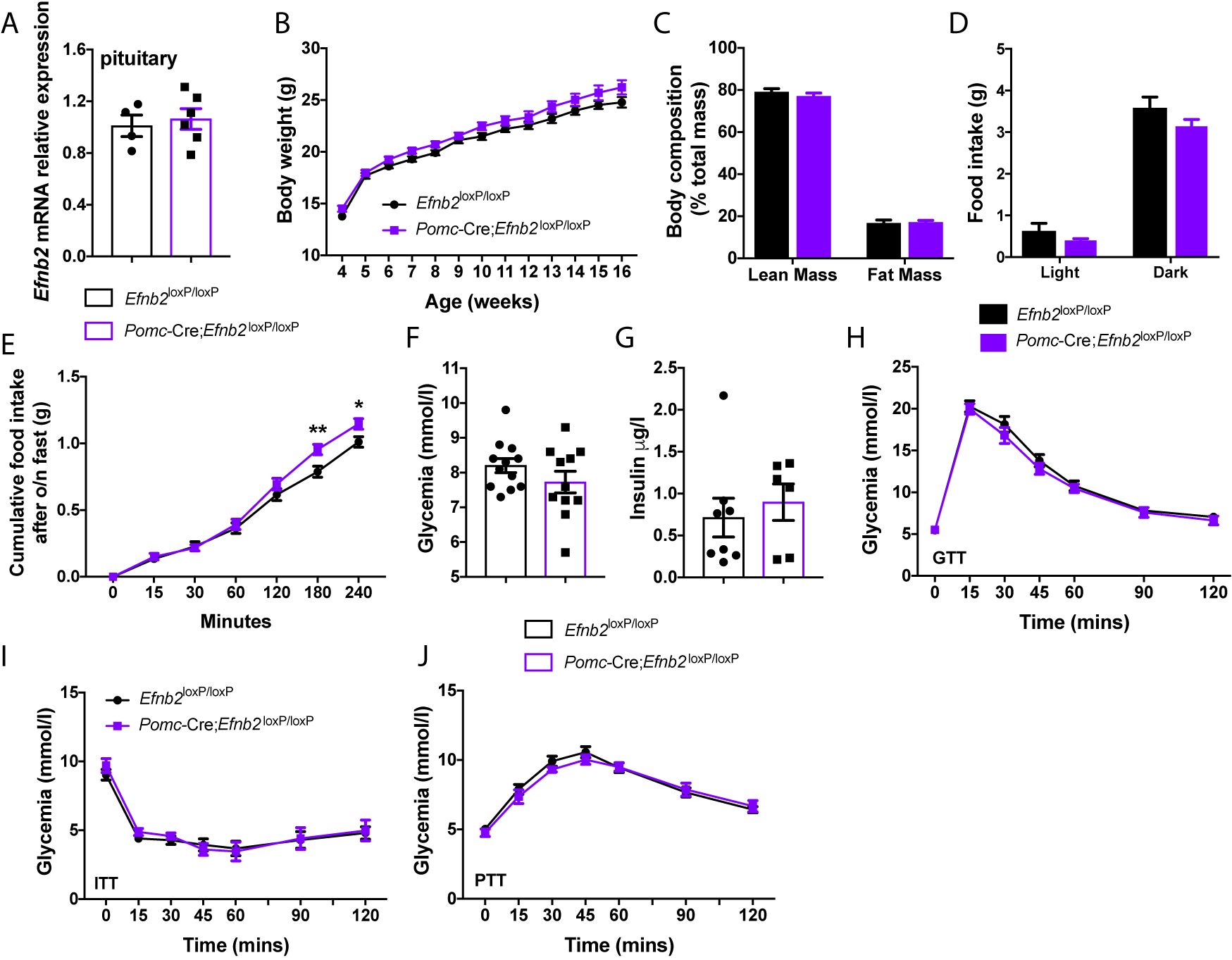

Supplement: S5 Fig — (A) Efnb2 mRNA relative expression in the pituitary of Efnb2loxP/loxP and Pomc-Cre;Efnb2loxP/loxP male mice (n = 4–6/group). (B) Post-weaning growth curve of Efnb2loxP/loxP and Pomc-Cre;Efnb2loxP/loxP female mice (n = 19/group). (C) Body composition of 16-week-old female mice (n = 6/group). (D) Food intake of 13–14-week-old female mice (n = 8/group). (E) Refeeding after overnight fasting of 13–14-week-old female mice (n = 11–15/group). (F) Basal glycemia of 8-week-old female mice (n = 11–12/group). (G) Basal insulinemia of 16-week-old female mice (n = 6–8/group). (H) Glucose tolerance test of 8–10-week-old female mice (n = 13–14/group). (I) Insulin tolerance test of 14-week-old female mice (n = 8–10/group). (J) Pyruvate tolerance test of 12–13-week-old female mice (n = 8–14/group). Data are shown ± SEM. Statistical significance was determined using 2-way ANOVA (B–E, H–J) and 2-tailed Student t test (A, F, G). *P ≤ 0.05 versus Efnb2loxP/loxP (E); **P ≤ 0.01 versus Efnb2loxP/loxP (E). The underlying data are provided in S1 Data. ANOVA, analysis of variance; POMC, proopiomelanocortin; SEM, standard error of the mean. (TIF) [file pbio.3000680.s005.tif]
