# Supplementary material for: EphrinB1 modulates glutamatergic inputs into POMC-expressing progenitors and controls glucose homeostasis
Source: PLoS Biol. 2020 Nov 30;18(11):e3000680. doi: 10.1371/journal.pbio.3000680 (PMC7728393; doi:10.1371/journal.pbio.3000680)
Supplement: S1 Table — (DOCX) [file pbio.3000680.s006.docx]

| *Sema3a* | *Sema4b* | *Sema5c* | *Netrin3* | *Efna2* | *Bdnf* |
| --- | --- | --- | --- | --- | --- |
| *Sema3b* | *Sema4c* | *Sema6a* | *Netrin4* | *Efna3* | *Ngf* |
| *Sema3c* | *Sema4d* | *Sema6b* | *Netring1* | *Efna4* |  |
| *Sema3d* | *Sema4e* | *Sema6c* | *Netring2* | *Efna5* |  |
| *Sema3e* | *Sema4f* | *Sema6d* | *Slit1* | *Efna6* |  |
| *Sema3f* | *Sema4g* | *Sema7a* | *Slit2* | *Efnb1* |  |
| *Sema3g* | *Sema5a* | *Netrin1* | *Slit3* | *Efnb2* |  |
| *Sema4a* | *Sema5b* | *Netrin2* | *Efna1* | *Efnb3* |  |
