# Supplementary material for: EphrinB1 modulates glutamatergic inputs into POMC-expressing progenitors and controls glucose homeostasis
Source: PLoS Biol. 2020 Nov 30;18(11):e3000680. doi: 10.1371/journal.pbio.3000680 (PMC7728393; doi:10.1371/journal.pbio.3000680)
Supplement: Attachment — Submitted filename: Response to Reviewers_PlosBiol_Croizier.pdf [file pbio.3000680.s009.pdf]

**We thank the reviewers for their comments. Our specific responses are detailed below in bold.**

Reviewer #1: The authors have responded satisfactorily to most comments. Yet, a few issues remain unresolved:

1) Line 145: The acknowledgement of P14-33 as an outlier suggests a distinction btw POMC->NPY and NPY->NPY, opposite to the authors' assertion.

**We thank the reviewer for this comment and have changed the text accordingly: "The principal component analysis (PCA) of the RNA-seq data reveals that POMC->NPY and NPY->NPY populations seem to be distinguishable but because of the outlier sample P14.33 NPY->NPY, this difference is not significant (S1A Fig). To facilitate our study, we only focused our attention on results obtained from POMC->POMC and NPY->NPY neurons."**

2) Given the absolutely minimal overlap of Pomc-GFP +ve and Pomc-Cre-tdTom +ve cells), how do the authors know that the Pomc-GFP transgenic mouse recapitulates any native Pomc expression at P14? It is not clear what the authors mean by "during postnatal day 14, the number of arcuate Pomc neurons is more important to that observed in adults".

**We only tried to give an explanation (in addition of the change in cell fate) of the minimal overlap between Pomc-progenitors and Pomc-GFP in adults. At postnatal ages, the absolute number of POMC neurons is higher to that observed in adults. Consequently, at these ages, the overlap between Pomc progenitors and POMC neurons is more important when compared to adults. But we agree with the reviewer that Pomc-Cre mouse model is not a perfect model to exclusively target POMC neurons.**

3) DAPI is conventionally blue. In Figure 1A (high magn.), nuclear staining is completely undiscernible. Images in Figures 4A, S1C are still missing DAPI. Figure 8C too.

**We chose the white color to illustrate the nuclear staining, as the blue color was too dark and was hiding the mRNA punctiform staining. Unfortunately, we did not have DAPI staining for the high magnifications. DAPI counterstaining was indeed performed on our sections as you can see in low magnifications but not acquired when using x63 objective because we quantified the vglut2+ inputs that were in contact with POMC somas.**
**Regarding figure S1C, I deeply apologize for this missing information probably due to mistake during the acquisition or saving of this picture. The counterstaining would have been helpful to better distinguish the arcuate neurons. However, we cannot take this picture as the RNAscope staining do not persist over time. We are confident in the way we quantified the number of Efnb1 and Efnb2-positive spots as Pomc-GFP cytoplasmic labeling was distinguishable.**

Nuclear staining in Fig. S4 is indicative of tissue deterioration.

**We agree that the tissue seems to be degraded, specifically in areas surrounding the pituitary, probably due to retrieval step during the in situ hybridization, but the mRNA staining does not seem to be altered.**

Reviewer #2: The authors have performed additional sutides which has addressed the Reviewers concerns in a satisfactory manner, which further strengthens the manuscript.

Reviewer #3: The authors have responded effectively to the majority of my comments and the comments of the reviewers. The manuscript is much improved. Unfortunately, the authors did not directly address my concerns about figure 3. I apologize if I was not clear - but my concerns remain. Here the authors show images of GFP labeled neurities and stain for vGlut2 a presynaptic marker. They show that there are decreases in this vGlut2 staining. These data would seem to suggest that in this figure, ephrin-B shRNAs are acting in the axon. However, for the rest of the manuscript, they claim that ephrin-Bs are acting postsynaptically. It seems that either the model system used in this figure is NOT appropriate for their studies (eg it looks at ephrin-B functions in AXONS, not dendrites) or ephrin-Bs are not functioning in axons in their studies. Based on the rest of their work, it seems likely that ephrin-Bs are postsynaptic in the POMC. This means that this assay is not a good
model for their system. If the effects of ephrin-B are postsynaptic, the authors must look at postsynaptic markers to validate their shRNAs. In addition, no rescue controls are shown for these tools.

**We agree with the reviewer comments and decided not to add these data in the manuscript. We therefore changed the text accordingly.**
