# Supplementary material for: EphrinB1 modulates glutamatergic inputs into POMC-expressing progenitors and controls glucose homeostasis
Source: PLoS Biol. 2020 Nov 30;18(11):e3000680. doi: 10.1371/journal.pbio.3000680 (PMC7728393; doi:10.1371/journal.pbio.3000680)
Supplement: Attachment — Submitted filename: Response to reviewers_plosBiol.docx [file pbio.3000680.s008.docx]

**Thank you for considering a revised manuscript. We have carefully considered comments made by the Reviewers and have spent time undertaking a series of additional experiments to refine our understanding of the mechanisms by which EphrinB1 and EphrinB2 modulate the glutamatergic inputs onto POMC neurons that play a key role in the regulation of glucose homeostasis. Our specific responses are detailed below in bold.**

REVIEWS:
Reviewer #1: In this manuscript, the authors attempt to assign a role of Ephrin B1 and B2 in the development of glutamatergic Pomc neurons of the arcuate hypothalamus. They provide evidence that glut +ve neurons of the paraventricular hypothalamus express ephrin receptors that may interact with Ephrin B1/2 of Pomc neurons to stabilize Glut termini and promote formation of glut termini by Pomc neurons. The authors also provide evidence that ablation of Ephrin B1 or B2 using the Pomc-Cre allele results in decreased glut termini of Pomc neurons, and has implications in glucose homeostasis (in the case of B1), and glucose homeostasis or impaired food intake that are sex specific. This is generally a well-written manuscript. There are, however, noticeable technical weaknesses, as well as weakness in correctly interpreting results. The PVH story is loosely connected, as the authors do not show localization of ephrin receptors in PVH neurons, and do not knock out the receptors to
show the downstream effect on Pomc neurons. Some of the staining is not convincing that makes conclusions questionable. In detail:

**We thank the reviewer for these comments. In this study, we attend to assess the role of EphrinB1 and EphrinB2 in the overall glutamatergic input pattern onto POMC-expressing progenitors (POMC^Prog^). We used the PVH as a model given the proportion of glutamatergic neurons innervating arcuate POMC neurons to study *in vitro* the influence of EphrinB1 and EphrinB2 on the number of glutamatergic terminals onto these neurons. We are sorry about the confusion and added this sentence in the main text. “We therefore decided to use the glutamatergic neurons of the PVH as a model to study the role of EphrinB1 and EphrinB2 in the control of the number of glutamatergic inputs into POMC neurons”.**

**We showed by *in situ* hybridization in Figure 2A that two receptors *Ephb1* and *Ephb2* were expressed in most of the glutamatergic PVH cells at P14. In addition, although we did not use histological approaches, we also showed that other known receptors for EphrinB1 et EphrinB2 were expressed during postnatal ages in the PVH (RT-qPCR, Figure S3).**

Line 75: Citations are missing.

**We apologize about the missing citations and have now added three citations in the text.**

Lines 70-76: Paragraph ends abruptly. Is text missing? Either way, this part should be re-written.

**We agree with the reviewer comment and have edited this paragraph.**

Lines 104-107: In the principal component analysis illustrated in Fig S1, it appears that NPY->NPY cells are vastly different from POMC->NPY cells, as p14-33NPY->NPY appears as an outlier. This is opposite to what authors claim.

**We thank the reviewer for this question. We agreed that p14 33 NPY->NPY is an outlier as he does not cluster with the 2 other NPY->NPY samples. We modulated our conclusion and accordingly changed the text: “POMC->NPY and NPY->NPY populations display high variability and overlap in the principal component analysis (PCA) of the RNA-seq data. Because of the outlier sample P14.33 NPY->NPY, we cannot distinguish POMC->NPY from NPY->NPY neuronal subpopulations (Figure S1A).”**

Regardless, FACS adds considerable manipulations and lengthy ex vivo incubation, hence making transcriptional profiling non-physiological. What was the viability percentage of cells after papain dissociation?

**We thank the reviewer for this comment and we would like to specify that the cells were sorted 1h to 1h30 after the brain dissection. We also analyzed the viability percentage in 4 of our P14 samples. These percentages vary from 91.8 to 95.7% (see table and figure below).**

| Samples | Viability % |
| --- | --- |
| P14 80 | 92.9 |
| P14 81 | 95.7 |
| P14 82 | 91.8 |
| P14 40 | 94.2 |

**Moreover, to make sure the right neuronal populations were sorted, we assessed the relative level of expression of both *Pomc* and *Npy* mRNA in each of the three neuronal subsets: POMC->POMC (red), POMC->NPY (yellow) and NPY->NPY (green) (see graph below). *Pomc* mRNA is only detected in POMC->POMC (left panel) and *Npy* mRNA is enriched in POMC->NPY and NPY->NPY when compared to POMC->POMC (right panel). For more clarity, we have now added the following graphs in Figure 1 and text in the manuscript.**

**“To validate the quality of the sort, we assessed by RT-qPCR the relative level of expression of *Pomc* and *Npy* mRNA in POMC->POMC, POMC->NPY and NPY->NPY populations. As expected, *Pomc* mRNA is highly expressed in POMC->POMC and undetectable in POMC->NPY and NPY->NPY neurons. On the contrary, *Npy* mRNA is enriched in POMC->NPY and NPY->NPY neurons when compared to POMC->POMC population.”**

If high, then it also poses as a confound regarding expression profiling and cell type survival. Do the FACS proportion of NPY->NPY, POMC->NPY and POMC->POMC cells recapitulate in vivo observations?

**The graphs shown above confirmed the efficiency and the quality of the sort as *Pomc* mRNA is enriched in POMC->POMC population and it is not detected in NPY neuronal populations. On the contrary, *Pomc* is depleted in NPY neuronal populations. However, our sort might not recapitulate the in vivo proportions of POMC and NPY neurons. By performing RNA-seq on these populations, we only seek to identify new factors involved in the glutamatergic synapse formation in these neurons, both POMC and NPY. In addition, we normalized our experiment by using the same RNA quantity from the 3 sorted neuronal populations, we sequenced a very high number of total reads (98-138M reads per library) and expressed our results based on the count of mRNA per million.**

Lines 112-113: Spell out what cells were enriched for Efnb1/2.

**We have now added a sentence to clarify which cells were concerned. “In particular, *Efnb1* and *Efnb2* were 5.2- and 16-fold enriched in POMC->POMC neurons when compared to NPY->NPY neurons, respectively”.**

Lines 116-122: Pomc-GFP +ve neurons and Pomc-Cre-tdTomato neurons minimally overlap (Padilla et al., Endocrinology 2012). Authors cannot compare distribution of Efnb1/2 in Pomc-GFP neurons with FACS/RNAseq data generated with the Pomc-Cre-tdTomato mouse.

**We agree with the reviewer comment that *Pomc*-cre;tdTomato+ cells encompass POMC neurons but also other populations such as NPY or Kiss neurons. We used this mouse model to identify genes putatively involved in glutamatergic inputs onto POMC neurons. Indeed, from the same tissue we sorted Pomc->POMC (red), Pomc->NPY (yellow) and Npy->NPY (green) neurons. Thus, the remaining red cells got rid of NPY neurons and are consequently enriched in POMC neurons even if it is not the only population to be present. We confirmed by histological approaches that POMC neurons (POMC-GFP+) themselves express *Efnb1* and *Efnb2* mRNAs. We also showed that NPY neurons express *Efnb1* and *Efnb2* (RNA-seq data). We do not and cannot exclude that Kiss+ neurons also express *Efnb1* and *Efnb2* as they also probably receive glutamatergic inputs. In this experiment shown in Figure S1, we only meant to assess whether or not POMC+ neurons could differentially express one of these gene as we performed bulk and not single-cell RNA-seq.**

**I also would like to emphasize that during postnatal ages (P14), the number of arcuate POMC neurons is more important to that observed in adults, limiting the tdTomato overlap with adult POMC neurons in Padilla et al. 2012. Please refer to Croizier et al. 2018, elife, Figure 2.**

**But to avoid any confusion in data interpretation, we changed POMC neurons for POMC-expressing progenitors (POMC^Prog^) in the text when referring to Pomc-tdTomato cells and used POMC neurons when referring to POMC-GFP cells.**

Line 133: typo

**We apologize for the typo and edited the text.**

Line 138: It is not very convincing that the mCherry +ve cells are also glutamatergic. Cells at the top left of the 50um image in Fig. S2B seem Vglut2 +ve but they are not mCherry positive. Moreover, the quality of the tissue seems compromised.

**We added DAPI staining in the picture. We cannot have a 100% overlap between PVH neurons and mcherry+ staining as 1) not every PVH neurons project onto POMC neurons, 2) the viral injections did not target every POMC neurons of the arcuate nucleus limiting the retrograde spread of the virus. However, all the mcherry+ cells display punctiform green vglut2 mRNA staining.**

Fig. 2A: Is this RT_PCR from total RNA from the whole PVH? Specify. What is the expression of these genes before or after glutamatergic terminals are formed?

**The RT-qPCR were performed on the whole neuroendocrine PVH that were microdissected from pups at several ages. We have now specified it in the main text. In adults, we observed the presence of both *Ephb1* and *Ephb2* mRNA (*in situ* hybridization, Allen Brain Atlas). The expression of these receptors is not surprising as they are also involved in synaptic plasticity.**

P56, Ephb1 mRNA, PVH

P56, Ephb2 mRNA, PVH

Fig. 2B: Image resolution is poor but it looks like Vglut2 mRNA staining is more convincing than in Figure S2B. How so? Images in Figure 1, Figure 2, Figure 4, Figure S1, S2, S4 require nuclear staining.

**We apologize for the poor quality of the picture probably due to the submission process. This picture has been acquired and saved on high resolution parameters and appears in high quality in illustrator format, however we increased again the resolution of the picture. Nonetheless, we do not exclude a differential expression between developmental ages and adult animals explaining the differences in term of number of colored dots.** **We added DAPI counterstaining images to help a better read of the picture.**

Fig.2D: what is depicted in yellow and what is in blue? Not clearly labelled

**We apologize for the lack of clarity and have now clarified what was depicted in blue (EphrinB1/B2) and in yellow (Eph receptors).**

Fig. 3: Method by which ARH/PVH cells were co-cultured in vitro is not described.

**We thank the reviewer for this comment. The method we used is detailed in the materiel and methods section but to allow a better understanding of the experiment we have added these sentences in the main text. “PVH and ARH were dissected from *Pomc*-eGFP mouse brain. PVH and ARH-containing cells were thus dissociated and both were co-cultured in the same well to enable the formation of neuronal networks (Figure 3A).”**

Again, Pomc-eGFP and Pomc-Cre-tdtomato lines cannot be interchangeably used because they label virtually different cells.

**We completely agree with the reviewer comment but by using Pomc-eGFP mouse model we seek to focus our study on POMC neurons and to compensate the weakness of the Pomc-Cre mouse model that do not only target POMC neurons but also NPY and Kiss neurons. We also changed POMC neurons for POMC-expressing progenitors (POMC^Prog^) when referring to Pomc-tdTomato cells and used POMC neurons when referring to POMC-GFP cells.**

**We have now added a paragraph is the discussion to raise the limitation of this mouse model.**

Lines 160-162: Did the authors look at localization of the receptors in PVH neuro termini? Why not knock down EphB1/B2 and look at Pomc glut. termini?

**We did not evaluate the localization of the receptors in PVH neuron terminals. We used PVH as a model to co-culture these neurons with ARH neurons to allow the development of a neuronal network. Indeed, we tried to only culture ARH neurons but the development of a neuronal network was absent or very limited.**

**We could have knock down EphB1/EphB2 in the PVH and bring specificity in our in vivo model. We decided not to do so as the PVH neurons are not the only glutamatergic neurons projecting onto POMC neurons. Our physiological effects could also be due to another excitatory source of neurons.**

Fig. 4: DAPI is missing.
Lines 174-176 and 216-218: Weak conclusions. The measurement of Efnb1/2 mRNA in the whole pituitary may have diluted out the lack of mRNA in the Pomc cells of the pituitary. Just because total pituitary expression is not affected, does not mean that the Pomc cells of the pituitary are not contributing to the phenotype.

**We thank the reviewer for this comment and agree with it. We have now added these sentences in the text. “However, we cannot exclude that POMC neurons expressed in the pituitary will not contribute to the phenotype of the mice lacking *Efnb1* in POMC-expressing progenitors.” “Again, we cannot exclude that POMC neurons expressed in the pituitary will not contribute to the phenotype of the mice lacking *Efnb2* in POMC-expressing progenitors.”**

Fig.6. Where are the images of glut termini on Pomc neurons?

**We now have added these pictures in Figure 8 panel C.**

Line 225: typo
**We have edited the text.**


Reviewer #2: The manuscript by Gervais and colleagues examines the role of EphrinB1 in Pomc neurons in the regulation of glucose homeostasis. Here, they report an enrichment of EphrinB members in Pomc neurons when glutamatergic inputs develop. While mice lacking Efnb1 in POMC neurons exhibit impaired glucose tolerance, in part through decreased glucose-induced insulin secretion these effects were not observed in mice lacking Efnb2 in Pomc neurons. Taken together, the authors conclude that ephrins control excitatory input to Pomc neurons to regulate glucose homeostasis.  Overall, the studies are well-designed and conducted and the data are of interest.

The primary finding is that mice lacking Efnb1 in POMC neurons exhibit impaired glucose tolerance, an effect associated with reduced glucose-induced insulin secretion.  The authors suggest that this is due to blunted vagus nerve activity.

Is pharmacological activation of the PNS sufficient to rescue the effect or does blockade of the PNS worsen glucose tolerance in WT mice, but not in KO mice?

**We thank the reviewer for this comment. It has been clearly established for decades that stimulation of the parasympathetic vagus nerve induces pancreatic insulin secretion (see review by Ahren 2000). Pharmacological activation and inhibition of the PNS would not be only restricted to pancreas and would provide confusing results. With respect to the reviewer, we decided not to perform these experiments.**

The current studies were conducted only in mice fed standard chow and both the energy- and glucose-homeostasis phenotype may be further exaggerated on animals subjected to a high-fat diet.

**We agree with the reviewer’s comment and we indeed fed males and females Pomc-Cre;Efnb2loxP/loxP and Efnb2loxP/loxP animals with high fat diet (Safe). After 6 weeks on high fat diet, the body weight was not changed between control and conditional mutant mice. However, we cannot conduct this experiment further as we had to sacrifice all of our experimental mice and most of our mice in stock due to the lock down of the institute and the COVID-19 situation.**

Is there evidence that mice lacking Efnb1 in POMC neurons exhibit reduced glutamatergic input using electrophysiology?
**We thank the reviewer for this very interesting comment that will surely increase the quality of the study. We performed electrophysiological recordings to assess the spontaneous excitatory postsynaptic currents (sEPSC) in arcuate POMC-expressing progenitors (Pomc-Cre;tdTomato-positive cells) localized in the dorsal and lateral parts of the ARH where most of POMC neurons are observed. Interestingly, we showed that the lack of Efnb1 in Pomc-expressing progenitors leads to a decrease of the amplitude and the frequency of the sEPSC. We made a new figure (Figure 6) to illustrate these data and added the following sentences in the results section.**

**“To determine whether the decrease of AMPAR and NMDAR subunits is associated with an alteration of the excitability of POMC^prog^, we recorded AMPAR-mediated sEPSC in tdTomato-positive cells found in dorsal and lateral parts of the ARH where most of POMC neurons are found using electrophysiology approach (Figure 6A). The lack of *Efnb1* in POMC^prog^ causes a decrease of the amplitude (Figure 6B and 6C) and the frequency (Figure 6B and 6D) of sEPSC, respectively”.**

The Discussion on POMC neurons in glucose-sensing is too speculative and not supported by the current studies.

**We agree that this paragraph was too speculative and probably not appropriate for this study. We therefore deleted it.**


Reviewer #3: In the manuscript by Dr Gervais and colleges the authors examine a mechanism for generating synaptic connections in the POMC and examine the impact of these connections on glucose homeostasis. Overall this is an interesting study that provides new information in an important area of research. There are a few issues that need to be addressed.

1.	The paper lacks clear explanation for how some experiments are conducted. For instance in Figure 3, it is unclear whether the eGFP labeled cells are the ones where ephrin has been knocked down.

**We thank the reviewer for this comment and we added a paragraph directly in the results section in addition of the methods section. The Ephrin known down has been conducted in the whole culture and not only on Pomc-GFP cultured cells. We do not exclude that Efnb1/Efnb2 has been silenced in PVH neurons as these mRNAs are expressed in PVH neurons during development (see graphs below).**

**In our *in vitro* model, as we co-cultured PVH and ARH neurons and knowing that EphrinBs are expressed in both nuclei, we cannot exclude a silencing of *Efnb1/b2* at both pre and postsynaptic levels. This is one limitation of the system. However, PVH neurons are exclusively glutamatergic and the majority of POMC neurons are GABAergic (Jarvie and Hentges, 2012).**

2.	In figure 3, to define synapses the authors need to stain for both pre (vGlut2) and postsynaptic markers (glutamate receptors or scaffolding proteins).

**We thank the reviewer for this comment. We tried not to use the term ‘synapse’ as we did not label the terminals with both pre and postsynaptic markers. However, we sometimes did use by mistake the term ‘synapse’ and have now changed it.**

3.	It is unclear whether the authors are proposing ephrin-Bs are located in axons or on the postsynaptic cell.

**We are sorry about the lack of clarity. EphrinBs are located on postsynaptic POMC cells. This information is summarized in figure 2D. We also have added the term ‘presynaptic’ when we cited the figure 2 in the text.**

For glutamatergic synapse formation in the CNS, ephrin-Bs are typically axonal. It appears in figure 3 that the green neurites shown are axons. The authors should determine whether this is the case by staining for tuj1 or other axonal markers.

**We thank the reviewer for this comment. Regarding the very low thickness of the neurites, it appears that they probably are axons rather than dendrites. Moreover, it has been shown on brain sections that on the contrary of AgRP neurons, arcuate POMC neurons do not display dendritic spines (Liu et al. 2012).**

**A few studies showed the role of postsynaptic EphrinB members in controlling synapse formation and plasticity. In our *in vitro* model, as we co-cultured PVH and ARH neurons and knowing that EphrinBs are expressed in both nuclei, we cannot exclude a silencing of *Efnb1/b2* at both pre and postsynaptic levels. This is one limitation of the system. However, PVH neurons are exclusively glutamatergic and the majority of POMC neurons are GABAergic (Jarvie and Hentges, 2012).**

This would help to clarify their model and story.

4.	The authors propose that in the ephrin-B knockout mice that there are reduced number of excitatory synapses. However, the authors only show a decrease in vGlut2 puncta. Given the model that changes in both AMPAR and NMDAR levels may be needed, the authors should stain for these markers in the ephrin-B knockout mice. This would help to determine whether the authors model is correct.

**We thank the reviewer for this comment. We have now quantified the number of mRNAs of *Gria1* (an AMPAR subunit) and *Grin1* (an NMDAR subunit) in POMC neurons by using the RNAscope approach (see graphs below). We observed a decrease of both *Gria1* and *Grin1* mRNA in arcuate POMC neurons in mice lacking *Efnb1* in POMC-expressing progenitors when compared to control mice. We added this information in the text and made a new Figure 5.**

**“To assess whether the reduced number of glutamatergic inputs into POMC^prog^ is associated with a decrease of glutamatergic AMPA and NMDA receptors, we labeled AMPA and NMDA subunits mRNA, *Gria1* and *Grin1*, respectively (Figure 5A). We thus quantified the number of *Gria1* and *Grin1* mRNA spots in POMC-expressing neurons in mice lacking *Efnb1* in POMC^prog^. As expected, we observed a 29% and 28% respective decrease of *Gria1* and *Grin1* mRNA spots in conditional mutant mice when compared to control mice (Figure 5B and 5C).”**

**Moreover, we also have recorded the excitatory postsynaptic currents (EPSC) in *Pomc*-Cre;tdTomato neurons in mice lacking Efnb1 in POMC-expressing progenitors and in control mice. The lack of Efnb1 leads to a decrease of the amplitude and the frequency of the sEPSC recorded in Pomc-expressing progenitors.**

5.	The sex dependent differences are interesting. The authors should address whether the pattern of expression of ephrin-Bs or the impact on vGlut2 puncta differs between male and female animals.

**We thank the reviewer for this comment. The first experiments we performed do not seem to show any differences in the expression of *Efnb2* in the ARH between males and females (see graph below, left panel), but these RT-qPCR were performed on the whole ARH, and not on POMC sorted cells.**

**To be more specific and to be as close as possible of our model, we thus quantified the number of *Efnb2* mRNA in *Pomc*-mRNA-expressing neurons in males and females at P14 (RNAscope). Interestingly, we observed an increase in the number of *Efnb2* mRNAs in females when compared to males (see graph above, right panel). Although very interesting, we prefer not to add these data in the manuscript as we aim to address the sexual dimorphism question in another project.**

Minor

The authors often make statements without references, particularly when describing the function of ephrins and Ephs. A careful review of the manuscript for missing references in needed.

**We agree with the reviewer and we apologize. We now have properly added references in the text.**
